# Enablers to successful patient and public involvement in Palliative Care Research: Qualitative perspectives of public members and Program Coordinators

**DOI:** 10.1177/02692163261450746

**Published:** 2026-05-25

**Authors:** Mollie Wilson, Jennifer Philip, Christine Hofmeyer, Avi Paluch, Stacey Panozzo, Peter Hudson, Anna Collins

**Affiliations:** 1 Department of Medicine, University of Melbourne, Australia; 2 Palliative Nexus, St Vincent’s Hospital, Melbourne, Australia; 3 Voices for Palliative Care, Australia; 4 Centre for Palliative Care, St Vincent’s Hospital, Melbourne, Australia

**Keywords:** palliative care, public involvement, consumer involvement, qualitative research

## Abstract

**Background:**

Despite policy and health service imperatives for public involvement to be embedded within palliative care research, this practice is not commonplace. Few empirical studies have sought to explore and evaluate models of public involvement for palliative care.

**Aim:**

To explore experiences of public involvement within palliative care, and to identify enablers for successful models of involvement.

**Design:**

An exploratory qualitative design was utilized. Semi-structured interviews were conducted via zoom or telephone, and interview transcripts were subjected to inductive thematic analysis.

**Setting/participants:**

Purposive sampling of 23 participants with experience of public involvement in palliative care or related areas, across different health institutions in Australia and the United Kingdom, including public members and public involvement program coordinators.

**Results:**

Twelve public members and 11 program coordinators described their experience with public involvement in a palliative or related healthcare setting. Three themes emerged relating to successful involvement (1) *Relationship building and maintenance*: opportunities to enhance team familiarity, early involvement, consistent point of contact, and inclusion of more than one public member. (2) *Clarity around goals of involvement*: flexible roles and processes, ongoing communication, formal recognition of public input. (3) *Training and support*: mentoring opportunities, upskilling for public members and researchers. These enablers promoted a collegial atmosphere that enhanced personal and collective experiences of public involvement.

**Conclusions:**

This study reveals enablers that potentially shape the extent and effectiveness of public involvement in palliative care. Integrating these enablers has implications for future models of public involvement in palliative care and potential for enhanced research outcomes.

**What is already known about the topic?**

- Public involvement can improve the quality and relevance of health research.
- There is limited understanding of the optimal strategies for public involvement in palliative care research.

**What this paper adds**

- Public involvement in palliative care research is enhanced through relationship building and maintenance, clarity around goals of involvement and training and support.
- There are important differences in the motivations and expectations of public members and program coordinators regarding public involvement.
- There is a need to balance the personal and relational elements of involvement with formalized processes.

**Implications for practice, theory or policy**

- Future public involvement models should focus on creating flexible, collaborative frameworks, that allow for ongoing dialogue and two-way learning between public members and researchers.

## Background

There is widening acknowledgment that patient and public involvement can improve the quality, relevance, and impact of health research.^1^ Public involvement (elsewhere referred to as consumer and community involvement) has been defined as research that is carried out ‘with or by members of the public, rather than to, about or for them’.^2^ While varying definitions exist, broadly public involvement requires an active partnership between researchers and public members who have a lived experience relevant to the phenomenon under study.^3^ The term ‘public members’ encompasses patients, potential patients, caregivers, individuals who use health or social services as well as people from organizations who represent service users.^2^ The terms ‘engagement’ and ‘involvement’ are often used interchangeably, but growing consensus suggests that ‘involvement’ is more reflective of the active and authentic contributions of public members.^4,5^ Most research funding in the UK is conditional on public involvement,^3,6^ and this is increasingly the case in other international jurisdictions including Australia.^7^

Public involvement closely aligns to the provision of holistic person-centered care, which is a core goal of palliative care.^8,9^ Yet, compared to other disciplines, there is limited research investigating the nature of public involvement in palliative care.^10,11^ While the features of involvement are not dissimilar to other fields, issues stemming from power dynamics, diversity, and emotional cost may be uniquely complex in this setting.^12^ People with palliative care needs may be considered ‘vulnerable’, both as research participants and research partners.^13^ Recruitment of public members is therefore delicate, given the emotional nature of the research subject, and the potential for causing distress.^14^ The same is true of the nature and level of involvement. Flexibility and accessibility have been identified as key priorities for public members who could not meet regularly or long term,^12^ as well as accommodations for other commitments, including treatment and caring responsibilities.^12^ Indeed, the potential emotional complexity has resulted in reluctance to engage with public members.^12^

At present, there is limited evidence outlining optimal strategies for public involvement in palliative care research, and even less demonstrating how to effectively measure impact. One review found eight main themes central to enhancing involvement: definitions and roles; values and principles; organization and culture; training and support; networking and groups; perspectives and diversity; relationships and communication; emotions and impact.^12^ Other themes derived from a qualitative interview study of participants involved at a single research institution in the UK included: building and maintaining relationships; flexibility of involvement; and finding the ‘right’ people.^6^ Further research is warranted to understand how the facilitators of optimal public involvement apply across different research contexts and settings, and to identify key factors in the development of new public involvement programs relevant to palliative care.

This study sought to explore the experiences of people involved in existing public involvement programs specific or related to palliative care across different health research institutions, and to explore perspectives on successful public involvement strategies in palliative care. This was conducted with a view to identify core features of optimal public involvement in palliative care, as a preliminary step in the development and implementation of a novel public involvement strategy for palliative care in Australia.

## Methods

### Design

In keeping with the study’s exploratory aims, this study utilized an exploratory qualitative design, to gain in-depth insights into participant’s experiences and perceptions of public involvement and enablers to successful public involvement. An interpretative phenomenological approach was adopted to understand participants’ personal perceptions and experiences of public involvement.^15^ This approach facilitated the consideration of public involvement strategies through the lens of individual experience and meaning.

### Co-design procedures

The study was co-designed with two public members who each have a lived experience of palliative care and palliative care-related public involvement projects.^16,17^ Slattery et al.^18^ define co-design as ‘meaningful end-user engagement in research design’ that ‘includes instances of engagement that occur across all stages of the research process and range in intensity from relatively passive to highly active and involved’. Co-design encompasses many different strategies for involving public members in research. The present study utilized a collaboration approach, whereby there was an ongoing partnership between two public members (CH, AP) and members of the research team. The public member co-investigators attended fortnightly investigator meetings for the duration of the project and provided oversight and guidance that informed all stages of the project. Specifically, public co-investigators: contributed to the study design and protocol development; co-developed and piloted interview guides; advised on recruitment channels; reviewed participant-facing information; attended three additional meetings to discuss and validate data interpretation and reviewed and edited the final manuscript.

### Participants

Participants were people currently involved in established public involvement programs specific to palliative care or a related healthcare field (e.g. oncology). Public involvement programs encompassed differing levels of involvement, including consultation, collaboration, and user-controlled research.^2^ Participants were ‘public members’ (eligible participants included people with malignant or non-malignant disease, informal caregivers and family members) or program coordinators (eligible participants included public involvement experts, researchers, clinicians, educators). Sampling focused on existing programs within an Australian context, and, to explore models from more established settings, one internationally recognized public involvement palliative care program in the UK. All participants were adults (>18 years), fluent English speakers, and able to provide informed consent. Written consent was completed via REDCap survey and verbal consent was recorded at the beginning of each interview.

### Recruitment and data collection

Program coordinators were purposively recruited via email, initially via networks of the project team and expanded via snowball sampling generated through interviews with other coordinators. Two coordinators were recruited from the UK, as representatives from an established public involvement palliative care program. Public members were identified and purposively sampled through palliative care peak bodies and research or health institutions across Australia. All public members were Australian.

Participants took part in semi-structured interviews conducted between December 2021 and April 2022 and were given the option to undertake these by telephone or Zoom. Interview questions were developed by two public members (CH, AP) and two researchers (SP, AC), to explore participants’ experiences in existing public involvement programs, enablers to successful involvement, perspectives on the evaluation of public involvement programs, and recommendations for future programs (see Appendices 1 and 2). The semi-structured designed allowed for flexibility and responsiveness, to explore participants’ unique experiences while ensuring key topics were covered.^19^

### Data analysis

Qualitative interviews were transcribed verbatim, and transcriptions were checked against audio recordings to determine accuracy. Data were subject to inductive thematic analysis,^20^ underpinned by an interpretative phenomenological framework.^15^ The framework allowed for an in-depth description of individual participant’s perceptions and interpretations of their own experiences, while accurately reflecting experiences across the entire dataset. NVivo 14 was used for data management and analysis.^21^ Analysis was conducted concurrently with data collection and collection ceased when data saturation was reached.

As outlined by Braun and Clarke,^20^ the process of inductive thematic analysis was conducted as follows: (1) data familiarization was undertaken by two researchers (SP and MW) and involved repeated transcript readings and note-taking for individual cases. (2) Initial codes were generated from each transcript and were subsequently discussed in regular investigator team meetings (CH, AP, MW, AC, JP). Initial codes emerged directly from the data and were not pre-determined. (3) Following these initial discussions, codes were manually grouped into themes that reflected both individual experience and broader patterns. The public member and program coordinator datasets were grouped separately and then compared to identify areas of convergence and divergence. Emergent themes and sub-themes were consistent in both datasets. (4) Themes were reviewed, and through further discussions, were validated by public member co-investigators (CH, AP) and three researchers (MW, AC, JP). Any differences in interpretation were resolved through discussion until consensus was reached. There were no notable disagreements to highlight. (5) Final themes were titled by the co-investigators. (6) Quotes were re-read, and the most illustrative quotes extracted as exemplars.

### Ethics

Ethical approval was obtained from the Human Research Ethics Committee at St Vincent’s Hospital Melbourne (HREC/78232/SVHM-2021-281016). Reporting followed the COREQ criteria for qualitative research.^22^

## Results

### Participant characteristics

A total of 23 participants participated in interviews (28–98 min), including 12 public members (eight female, four male) and 11 program coordinators (all female). All public members were previous or current carers for a person with advanced illness (cancer or dementia) who had received palliative care. Public members had a vast range of experience in public involvement activities, including membership on advisory boards and project steering committees, short-term consultations on aspects of research project development, consultation for website content, development and testing, graduate training sessions, community volunteering and the facilitation of carer support groups (Table 1).

**Table 1.** Characteristics of program coordinators and public members.

Characteristics	Program coordinators (*n* = 11)	Public members (*n* = 12)
Gender		
Male	0	4
Female	11	8
Age		
36–45	4	0
46–55	5	1
56–65	2	4
66–75	0	5
76–85	0	2
Years of experience as a public member		
<1 year	-	1
1–2 years	-	1
3–4 years	-	5
5–6 years	-	2
7–8 years	-	1
More than 10 years	-	2
Setting of employment		
University institution	4	-
Peak body	3	-
Community palliative care	2	-
Non-for-profit organization	2	-

### Overview

Three overarching themes emerged as shared enablers to successful public involvement in palliative care: relationship building and maintenance; clarity around goals of involvement; training and support. The associated sub-themes identified specific techniques and strategies to achieve each overarching theme. The themes and sub-themes (Table 2) demonstrated what was working well in some contexts and raised areas for improvement in others. While the themes and sub-themes were consistently found in both the public member and program coordinator datasets, there were some differences in the ways that participants made sense of certain themes. Put simply, the reported experiences were broadly similar, but the meanings attributed to these experiences differed by cohort.

**Table 2.** Themes and subthemes.

Theme	Sub-theme
Theme 1. Relationship building and maintenance	Subtheme 1a. Opportunities to enhance team familiarity
	Subtheme 1b. Consistent point of contact
	Subtheme 1c. Inclusion of more than one public voice
	Subtheme 1d. Early involvement of public members
Theme 2. Clarity around goals of involvement	Subtheme 2a. Clear, yet flexible role parameters
	Subtheme 2b. Opportunities for ongoing feedback regarding involvement
	Subtheme 2c. A personal approach to remuneration
Theme 3. Training and support	Subtheme 3a. Peer mentoring and support
	Subtheme 3b. Training for public members
	Subtheme 3c. Training for researchers

Although experiences of public involvement were overwhelmingly positive, when these themes were not well addressed, public members sometimes perceived their involvement was inauthentic or curtailed. Involvement felt like ‘box-ticking’ when consultations were brief, at the end of research projects, or when public members felt dismissed by researchers. The latter produced power imbalances between public members and other members of the research team, leading to frustration, decreased confidence, and disengagement of public members, thus limiting the effectiveness of involvement.

“I think whenever you put [public members] in with highly trained professionals, there's a power imbalance. And depending on those people's personalities, they can either wreck your first experience or support it.” (Public member 10)

Program coordinators also noted the potential imbalances in public involvement, however, their focus remained on the strategies that could prevent these imbalances, rather than the personal impact of them.

### Theme 1. Relationship building and maintenance

All participants highlighted the role of relationships in facilitating productive, collaborative partnerships between public members and researchers. While *co-design* and *early* public involvement were emphasized throughout the interviews, they contributed to the broader theme of relationship building, as they strengthened a sense of camaraderie between members of the research team. Stronger relationships were fostered by opportunities to enhance team familiarity, consistent points of contact, inclusion of more than one public member, and timing of involvement.

#### 1a. Opportunities to enhance team familiarity

Public members spoke often of the importance of ‘getting to know’ the research team, but the mechanisms by which this occurred were sometimes unclear. Personal familiarity with the researchers increased team bonding and development, which, in turn, raised public members’ sense of ownership and investment in the project. Coordinators echoed the importance of a relational atmosphere but attributed this primarily to group size. The size of a research group shaped group dynamics and the level of familiarity between researchers and public members. Smaller groups were reported to create an environment that was more conducive to collaboration, open communication, and stronger connections between the team.

“I knew the group much better because it was only about 10 people. So, 10 versus 80. . . And so, the relationships were slightly different.” (Program Coordinator 07)

#### 1b. Consistent point of contact

Similarly, both participant cohorts discussed the importance of establishing a point of contact who could facilitate dialog between researchers and public members. This person, usually a chairperson or coordinator, was familiar to all team members, and accessible outside of scheduled meeting times. During meetings, this person would ensure that public members felt confident to speak up, and all voices were heard and valued. When there was staff turnover and the coordinator or chairperson changed frequently, public members were more likely to feel disconnected from the team and project.

“Usually, the chairs are very proactive in saying, "Phil or John, what do you think of this?" So, they're actually respecting that you are there. They know that you can't provide the clinical information and the technical and the data information, but they make you feel engaged by specifically asking you about areas or if you have a comment to make.” (Public Member 05)

#### 1c. Inclusion of more than one public voice

Relationship dynamics were strengthened by a more balanced ratio of public members to researchers, or at the very least, the involvement of more than one public member. When the public member was the only public representative, they were more likely to perceive power imbalances within the research team with associated reduced ability to influence decisions made.

“I’m on a couple of committees in the local health district where I’m the only [public member]. . . And you feel that your voice won’t be heard as well or as much. And you tend to be more reluctant to say things.” (Public Member 05)

In contrast, with the inclusion of more than one public member, public members could ‘bounce off’ one another with increased confidence. Witnessing the contributions of other public members activated new ideas and modes of involvement. Similarly, program coordinators recognized their role in facilitating collaborative discussions *between* public members, rather than solely between public members and researchers. One coordinator described best practice as involving *at least* two public members.

“The fact that [the public members] all know each other and it's a small group is helpful. They all bring forward their experiences to share with each other, so they're learning from each other.” (Program Coordinator 01)

#### 1d. Early involvement of public members

Relationships and project investment were improved by co-design strategies and early public involvement, for example, at the project design phase. When public members were brought into a research project at a later stage (i.e. dissemination of results) and for a predetermined purpose (i.e. meeting grant guidelines), involvement felt less genuine. Comparatively, when public members had the opportunity to inform the research in its early stages (i.e. supporting ethics applications, co-designing outcome measures), they felt more invested.

“The main problem with people working with [public members] is that they bring them in too much at the end. . . They've already got their answer, and they're sort of just wanting to say that that people are giving their answers. So, if you are wanting it to be real, you've got to get them in early.” (Public Member 02)

### Theme 2. Clarity around goals of involvement

#### 2a. Clear, yet flexible role parameters

Public members found their involvement to be most effective when they had a clear idea of why the researchers were seeking public involvement, the intended outcome of involvement, and the expectations around their role. This was often framed as a barrier by the public cohort, who admitted to feeling confused or misled about the parameters of involvement.

“Because there isn't a lot of clarity around the role, I probably go in a bit more cautiously than I should. I hold back a little.” (Public Member 06)

While participants wanted clear role parameters, they also valued a degree of flexibility. Given the nature of palliative care, flexibility was crucial to accommodate personal commitments and the changing needs of patients and carers. Flexibility in recruitment processes was also emphasized by both cohorts, with consideration of the changing nature of illness and grief.

“In palliative care particularly, the timing's important. Because the grief period can be very long. It's a bit hard to approach someone who recently lost someone who's been through palliative care, to see what they'd like to contribute.” (Public Member 05)

#### 2b. Opportunities for ongoing feedback regarding involvement

Role clarity was strengthened by ongoing feedback and communication between public members and researchers, outside of scheduled meetings. Interestingly, while both cohorts emphasized the value of feedback, they highlighted disparate reasons for its importance. Coordinators saw feedback as an opportunity to keep public members informed about the status of the research project. Feedback was also an opportunity to open dialog about the nature of public involvement, and why public member recommendations were taken on board, or not. This helped to clarify role parameters and manage expectations.

“So, it's a whole process that we want to bring them along the whole way. Even if they're not participating in all activities, we're letting them know everything that's going on with the project.” (Program Coordinator 10)

For public members, feedback was portrayed as a method of gaging impact. When researchers acknowledged public contributions, they felt that their input had made a difference. In this sense, ongoing and constructive feedback also counteracted potential barriers to involvement, such as perceptions of involvement as inauthentic.

#### 2c. A personal approach to remuneration

While coordinators did see feedback as an opportunity to recognize public input, they preferred formalized recognition, usually through remuneration. Paying public members for their input was mentioned frequently by coordinators, who considered remuneration to be a key process in standardizing role parameters. Payment could also alleviate power imbalances, ensuring that all team members were appropriately reimbursed for their time.

“. . . to really reiterate to our [public members], yes, you want to give back, but you should not be the only person in the room not paid to be there. Just because you want to give back, doesn't mean you have to volunteer every aspect of your life. Your time is your time.” (Program Coordinator 01)

In contrast, remuneration mentioned less frequently by public members. The recognition that public members sought was through feedback that acknowledged their contributions as useful and meaningful. For some public members, in fact, payment could be uncomfortable as it did not reflect their motivation for involvement.

“I know a lot of people feel that they need to give people money for their time or some sort of remuneration, but I've never felt that I wanted that. Like, I do it because I want to contribute.” (Public member 10)

### Theme 3. Training and support

All participants found public involvement-specific training to be helpful, as it could alleviate perceived power imbalances between public members and other members of the research team. Participants recognized the need for training that ideally addressed several facets of involvement, including public speaking, media training, research and health literacy, and informal mentoring with other public members.

#### 3a. Peer mentoring and support

Public members frequently discussed the value of being around other public members in an informal mentoring context. This allowed public members to build rapport and also facilitated learning from someone who had relevant and valuable experience. Although not formalized training, public members reported increased confidence after working with other public members. Program coordinators similarly recognized the advantages of buddy systems and informal learning between public members.

“But just that confidence that you get. . . if someone buddies up with you who has a bit more experience than you, that's encouraging.” (Public Member 07)

#### 3b. Training for public members

Both informal mentoring and formal induction sessions were most valuable when embedded early. When training occurred at the beginning of involvement, public members felt better equipped to perform the tasks expected of them and to contribute in the most meaningful way. Further, this helped to clarify role parameters and goals of involvement.

Informal upskilling was another helpful method to embolden public members and provide them with the appropriate tools to engage effectively. Rather than spending time and resources on large, formal training sessions, program coordinators discussed the utility of informal one-to-one sessions for relevant projects.

“I did an informal one-to-one session with her, talking about coding and all that sort of thing. . . And I think that’s probably one of the most common ways that we do that upskilling. . . is in response to needs on particular projects, and what people are interested in and want to get involved with.” (Program Coordinator 04)

#### 3c. Training for researchers

Participants emphasized the utility of researcher training and upskilling. Participants felt that researcher training was critical to ensure that researchers (a) understood the value of embedding lived experience in research, (b) were kept up to date with best practices of public involvement, and (c) had the tools to engage with public members in a sensitive and appropriate way.

“We actually get regular clinical supervision to be able to talk through some, how we help, how we deal with the [public members]. That's what really, really helped us. With what we learned from that clinical supervision, we can put into practice when we speak to some of those [public members].” (Program Coordinator 11)

## Discussion

### Main findings

The current study explored experiences and enablers of successful public involvement from the perspective of public members and program coordinators presently involved in research programs in Australia and the UK. Three key enablers emerged, with corresponding strategies: relationship building and maintenance; clarity around goals of involvement; training and support.

### What this study adds

This study identified some divergence in the perspectives of program coordinators and public members, that should be considered in future public involvement models. For example, while coordinators expressed a desire to systematize and formalize involvement processes, public members often prioritized the personal and relational aspects of involvement. The desire to systematize public involvement likely stems from the motivation to legitimize and promote the frequency of involvement in research (e.g. public involvement as a funding requirement). This may reflect an attempt to efficiently and rationally manage complex human activity as well as protect the public members via formal organizational processes.^23^ However, such processes may diminish the creative opportunities that arise from genuine collaboration. In a commentary on the learning outcomes of public involvement, Staley and Barron argue that public involvement should not be treated as an ‘intervention’ needing an evidence base, but rather, as ‘conversations that support two-way learning’.^24^ They suggest that attempts to standardize involvement processes ‘as ‘methods’ and to objectify the outcomes “may be akin to forcing a square peg in a round hole’”. An overly systematized approach to public involvement may inadvertently result in inflexible processes that undermine the rich human elements of authentic involvement. There is a need therefore to balance the personal motivations of public members with the formal processes that underpin involvement. Public involvement cannot be a strictly top-down, fixed process; rather, it should be characterized by ongoing dialog and two-way learning, to progressively refine and enhance the involvement model.

This study underscored the critical importance of trusted relationships across all members of the research team.^6,25–27^ For public members, sharing deeply personal and emotional stories relating to one’s lived experience requires trust. Genuine relationships were characterized by a sense of equality amongst team members, and mutual investment in research projects. However, in interviews with researchers and public members, Green and Johns found that equal relationships and ‘sharing power’ were often incompatible with the hierarchical nature of academic environments.^28^ They revealed that public members were often slotted into a ‘pre-existing structure’, rather than having autonomy over the scope of their own involvement. This experience was echoed by public members in the present study. Here, we note a tension between relationship building and clarity around goals of involvement. While clarity is important, the goals and scope of involvement should be collaboratively negotiated between team members and not predetermined by coordinators or institutions.^29^ As Pearce suggests, guidelines that truly wish to fulfill the aim of genuine relationship building must also acknowledge ‘how choice, agency and identity are relationally formed’.^30^

### Future directions

The themes and sub-themes raise several practical approaches for the development and implementation of future models of public involvement in palliative care. Building on our findings and existing public involvement guidelines, we have extrapolated a set of suggested approaches, presented in Table 3. These are preliminary and practical steps that may be considered from both institutional/strategic and individual/relationship levels (e.g. early recruitment and induction, consistent points of contact). These suggested approaches were co-developed and refined with our public member co-investigators and have been mapped to published public involvement guidelines and frameworks for health research (including NIHR,^25^ INVOLVE,^2^ and recommendations from the RAPPORT study^3^). However, they remain an interpretative synthesis of the present findings rather than formally validated guidance. To ensure rigor and acceptability across diverse settings, we recommend that future projects formally validate and refine these suggested approaches through staged stakeholder engagement, structured consensus methods (e.g. Delphi) and local co-design activities. As Greenhalgh et al.^17^ have suggested, a single, one-size fits all framework for public involvement may be less useful than resources and principles that have been locally co-designed.

**Table 3.** Suggested approaches for effective public involvement in palliative care.

Theme & Sub theme	Approaches: Individual level	Approaches: Institutional level	Alignment with existing guidelines
**(1) Relationship building and maintenance**			
(a) Opportunities to enhance team familiarity(b) Consistent point of contact(c) Inclusion of more than one public voice(d) Early involvement of public members	• Allocating time at the beginning of research meetings for informal conversation and rapport building.• Allocated program coordinators or research fellows who provide consistency and familiarity, as a single point of contact throughout the research project.• Skillful meeting chairs (or public member co-chairs) who can ensure that all members have opportunities to contribute.• During recruitment and induction, ensure time to discuss and understand the individual public member’s motivation for involvement in research.• Maintaining relationships with public members beyond the lifespan of individual projects.	• Pubic involvement research group sizes should not exceed 10–15 people.• Identified professional development opportunities for public members, including leadership positions and membership in investigator teams.• Accessibility of emotional support systems for public members, including peer-mentoring and debriefing.• Recruitment strategy designed in collaboration with public members. Recommended recruitment intake of public members at the same time (e.g. annually or bi-annually).• Timing of involvement: public involvement groups should meet on an ongoing basis. Ideally, involvement within specific research projects should begin prior to grant writing.	• RAPPORT: development of relationships over time (e.g. dedicated public involvement coordinator, benefits of small, stable group sizes).^3^• INVOLVE: recommends named contact person.^2^• NIHR: emphasizes early and sustained engagement and co-designed recruitment.^25^• Mitchell et al.’s^26^ framework for equitable public involvement in palliative care: relationships must be established and maintained over time.
**(2) Clarity around goals of involvement**			
(a) Clear and flexible role parameters(b) Opportunities for ongoing feedback regarding involvement (c) A personal approach to remuneration	• Reflexive and collaborative role development with ndividual public members• Developing personalized remuneration plans. All public members offered financial compensation if they would like it, but also, professional development opportunities or other incentives that align with individual preferences.• Initial discussions during induction to address time commitments, and the potential emotional burden of involvement.• Involvement of several public members can mitigate the burden on individuals by sharing responsibilities.	• Flexibility embedded within involvement strategies (e.g. hybrid blend of face-to-face and online meetings, options for smaller working groups)• Outline purpose of public involvement at recruitment stage.• Implementation of regular feedback sessions (e.g. annual review meetings, or 10-min check-ins following public involvement activities).• Feedback mechanisms embedded via Terms of Reference, with regular opportunities for review.• Levels of involvement (from brief consultations to ongoing collaborative partnerships) should be reflective of the type of project, with opportunities for public members to be involved at all levels.	• RAPPORT: shared understanding of the purpose, role and structure of public involvement.^3^• GRIPP2: report on the aim of public involvement in the study.^31^• INVOLVE: transparency around the aims and scope of public involvement (e.g. co-developed role descriptions).^2^• NIHR payment and recognition guidance.^25^• Mithcell et al’s equitable public involvement framework emphasize choice and flexibility^26^
**(3) Training and support**			
(a) Peer mentoring and support(b) Training for public members(c) Training for researchers	• Establishment of peer mentoring networks where experienced public members can mentor newcomers, providing practical advice, emotional support and encouragement.• Public members encouraged to identify training opportunities that would be of value.	• Induction pack for public members to include: cover letter and introduction to organization, terms of reference, public member role description, remuneration process.• Training programs that incorporate the lived experience voice or are co-facilitated by a public member.	• INVOLVE: training and support^2^• Cancer Australia National Consumer Framework: highlights the importance of building capacity of public members through training and support.^32^
		• Public member training programs should cover research methodologies, effective communication skills.	
		• Researcher training programs should emphasize the benefits of integrating lived experience in research and effective communication skills.	
		• Creation of resource hubs that provide easy access to training materials, guidelines and best practice documents.	

### Strengths and limitations

A notable strength of this study was its co-design approach, involving an ongoing partnership of public members and palliative care researchers. This methodology ensured that the lived experience perspective was embedded at all stages of the project.

Despite advertising through multiple organizations, all public members were carers, and no public members with a lived experience as a patient or person living with a malignant or non-malignant disease were recruited for the study. The unique demands and experiences of patients with palliative care needs involved in public involvement projects are essential to fully understanding optimal public involvement. The research teams personal experiences with public involvement (as both researchers and public members) and familiarity with the subject matter, could have inadvertently shaped data interpretation and interview directions. To mitigate this potential bias, an inductive thematic approach was employed to ensure that themes emerged directly from the data. Additionally, the suggestions and enablers raised by participants in this study were collected from two international settings with well-developed palliative care services, and as such, may not be generalizable to other less resourced settings. The suggested approaches are preliminary and will require further developmental work and evaluation including acceptability, feasibility and utility.

## Conclusions

This study identifies enablers to successful public involvement in palliative care. Instead of making assumptions about the vulnerability of those with palliative care needs, researchers should strive to create flexible and accommodating involvement parameters that enable diverse, individualized levels of involvement. Future programs should allocate time and resources to relationship building, co-designed goals, and support systems.

## Appendix 1

### Interview guide – Consumer representatives in palliative care

1. Can you tell me about your experience as a consumer linked with palliative care [research]?
  a. How did it come about?
  b. What things have you been involved in?
  c. From your experience what are the things made possible / accomplished when consumers are involved with palliative care / palliative research?
  d. What does it look like when it works really well – the team, the research itself, other areas?
  e. What does it look like if it doesn’t work so well? Or can you imagine what might be limits/barriers?
2. Who do you work with most in the palliative care [research] team?
  a. What do you look for in that person/ those people?
  b. What makes it easier?
3. Do you feel your involvement has made a difference so far?
  a. Can you share an example of something that has changed because of your involvement? How/why?
4. One of the things we have been thinking about is how we know (or show or indeed measure/evaluate) that consumer involvement has made a difference to palliative care [studies/research]. What are your thoughts on this? Can you share an example?
5. Do you have any other thoughts or recommendations for improving the involvement of patients and public (consumers) in palliative care?

**Thank you so much for participating and generously sharing your thoughts and experiences today.**

## Appendix 2

### Interview guide – Palliative care researchers, educators and clinical service leads

**A. Exploring experiences of public involvement in palliative care**
**1. Can you tell me about your experience in working together with public members in research/education/clinical services?**
  a. Can you tell me about how this way of working developed for you?
  b. What are the area(s) of public involvement, and what has been accomplished as a result?
  c. Do you think this involvement has made a difference? If so, can you explain how?
**2. What does public involvement look like when it works really well – the team, the research itself, other areas?**
  a. What needs to be in place to enable this?
**3. What does it look like if it doesn’t work so well? What are some of the downsides or barriers to public involvement?**
  a. Can you share an example of where public involvement did not work as expected?
  b. Have there been any times when you have been unable to engage public members in your projects?
**4. Which areas of the research process are public members more involved with, and why do you think this is?**
  a. Design? Dissemination? Presenting? Evaluation? Any others?
  b. Are there any areas you would like them to be more involved in? How could we achieve this?
**5. How do you acknowledge/recognise their contribution? Feedback? Payment? Any others?**
  a. Do you feel this is an effective recognition for the public member themselves?
  **B. Impact of consumer & community involvement**
**6. How do you judge whether public involvement has made a difference to your study?**
  a. Can you give me any examples of how you have changed your study because of public involvement?
  b. Do you evaluate the impact of public involvement at the end of the project? If so, how?
  c. Does it improve the relevance and/or quality of your research?
  d. Any other impact that public involvement has led to? For example, improving public involvement strategy? Improving communication with the public?
**7. Has engaging with public members had an impact on you?**
  a. Do you feel confident about engaging with public members within your research? Are you more interested to incorporate it their involvement in your next project?
**8. Do you feel that public involvement is embedded within your organisation? If so, how?**
  **C. Future needs & development**
**9. What training and/or information surrounding CCI would be beneficial to you and your research?**
**10. Do you have any other thoughts or recommendations for how we can improve CCI in palliative care?**
